# Exploring Risk Factors for Follicular Lymphoma

**DOI:** 10.1155/2012/626035

**Published:** 2012-09-18

**Authors:** Alexander J. Ambinder, Pareen J. Shenoy, Neha Malik, Alison Maggioncalda, Loretta J. Nastoupil, Christopher R. Flowers

**Affiliations:** Winship Cancer Institute, Emory University, N.E. Building B, 1365 Clifton Road, Suite 4302, Atlanta, GA 30322, USA

## Abstract

Follicular lymphoma (FL) is an indolent malignancy of germinal center B cells with varied incidence across racial groups and geographic regions. Improvements in the classification of non-Hodgkin lymphoma subtypes provide an opportunity to explore associations between environmental exposures and FL incidence. Our paper found that aspects of Western lifestyle including sedentary lifestyle, obesity, and diets high in meat and milk are associated with an increased risk of FL. Diets rich in fruits and vegetables, polyunsaturated fatty acids, vitamin D, and certain antioxidants are inversely associated with FL risk. A medical history of Sjogren's syndrome, influenza vaccination, and heart disease may be associated with FL incidence. Associations between FL and exposure to pesticides, industrial solvents, hair dyes, and alcohol/tobacco were inconsistent. Genetic risk factors include variants at the 6p21.32 region of the MHC II locus, polymorphisms of the DNA repair gene *XRCC3*, and UV exposure in individuals with certain polymorphisms of the vitamin D receptor. Increasing our understanding of risk factors for FL must involve integrating epidemiological studies of genetics and exposures to allow for the examination of risk factors and interactions between genes and environment.

## 1. Introduction

Non-Hodgkin lymphoma (NHL) represents a heterogeneous group of malignant lymphoproliferative diseases that vary significantly in their biological and clinical characteristics. As a group, NHL ranks as the seventh leading cancer diagnosis in the United States (US), and the American Cancer Society estimates that in 2012 there will be 70,130 incident cases of NHL and 18,940 deaths attributed to the disease [1]. NHL occurs more commonly in men than in women, in Caucasians than in Blacks, and in the elderly, though these broad trends do not necessarily reflect the epidemiology of individual subtypes. In the 1970s and 1980s, the epidemiology of NHL was marked by a dramatic 50% increase in incidence, concurrent with the rise of the HIV/AIDS epidemic and significant improvements in the lymphoid neoplasm classification system. Though these developments along with the increased prevalence of immunosuppressive therapies certainly contributed to this trend, the increased incidence has yet to be fully explained [2, 3]. Recent efforts to understand the etiologies of NHL have thus begun to shift focus to individual subtypes. Follicular lymphoma (FL) is one such subtype that has been studied extensively due to its prevalence, distinctive histology, and relatively preserved classification schema. 

### 1.1. Biology

FL is a malignancy of germinal center B-cells that is commonly associated with the inappropriate activation of the protooncogene, *BCL2*, found on chromosome band 18q21 [4]. *BCL2* belongs to a family of proteins which governs the mitochondrial apoptotic pathway. The dysregulation of the gene is highly antiapoptotic and is thought to play a crucial role in the pathogenesis of FL. Most commonly, *BCL2* is activated through the *t*(14; 18) chromosomal translocation, which juxtaposes *BCL2* and the IgH locus. Other less common translocations associated with the disease include *t*(2; 18) and *t*(18; 22), achieving similar dysregulation of the *BCL2* gene through juxtaposition with the IgK and IgA loci, respectively [4]. These translocation events are thought to occur during V(D)J recombination in the maturation of B cells. Nevertheless, *BCL2* activation is not sufficient to produce malignancy, since *BCL2* activation has also been shown to be present in either circulating B cells in healthy individuals and in possible precursor lesions including intrafollicular neoplasia/in situ FL [5–7]. Increasingly, it is thought that this is only one of many steps leading to oncogenesis. 

### 1.2. Epidemiology

FL is the second most common subtype of NHL, accounting for approximately 20–30% of all NHL cases. Between 1992 and 2001, incidence rates for FL did not change significantly, though incidence rates did increase by 1.8% in the elderly population [8]. FL is more common in Caucasians than in Blacks or Asians, and unlike other NHL subtypes, the incidence rates in men and women are similar. Although Blacks experience a lower incidence of FL, the average age of diagnosis in blacks is approximately 10 years younger than in Caucasians [9]. Furthermore, while survival in patients with FL has improved in recent years, these gains have been less pronounced in black populations [10]. In Caucasians, the 5-year relative survival rate (RSR) has increased from 52% in 1987–1989 to 71% in 2001–2007, while in Blacks, the 5-year RSR over these same time intervals were 46% and 62%, respectively [1]. 

### 1.3. Classification

The significance of these and other epidemiological trends in FL has been clarified by the continual evolution of the NHL classification systems. In 1997, the NHL Classification Project evaluated a recent iteration of the classification system proposed by the International Lymphoma Study Group, incorporating histological, clinical, immunophenotypic, and molecular/genetic data into its classification scheme [11]. This retrospective cohort study included 1,403 diagnosed cases of NHL and found improved diagnostic accuracy up to 85% with the incorporation of this new data and better interreviewer reliability. In 2007, the International Lymphoma Epidemiology Consortium (InterLymph) devised a hierarchical classification scheme that incorporated elements of the 2001 World Health Organization (WHO) Classification of Lymphoid Neoplasms and the International Classification of Diseases for Oncology (ICD-O, third edition), as well as a method for reclassifying disease that had previously been classified using the Working Formulation, Revised European American Lymphoma Classification, and ICD-02 [12]. This was updated in the 2008 WHO classification and has recently been incorporated by epidemiological researchers and surveillance programs such as the Surveillance Epidemiology and End Results (SEER) registry [12, 13]. These modifications to improve classification and surveillance facilitate the identification of risk factors and exposures that contribute to the development of each NHL subtype. FL is one subtype that has been more carefully studied for associated risk factors and exposures as a result of its relative prevalence. This paper surveys the literature to date on risk factors and associations with the development of FL.

## 2. Risk Factors for Follicular Lymphoma Incidence

### 2.1. Diet and FL

The dietary risk factors associated with FL are summarized in Table 1. In a 2005 Italian study, Polesel et al. found significant associations between diet and the incidence of NHL [14]. Using a validated food-frequency questionnaire (FFQ), researchers assessed the diets of 190 NHL (31 FL) patients. The data suggested an inverse relationship between linoleic acid, a polyunsaturated fatty acid (PUFA), and NHL risk (odds ratio (OR) = 0.6, 95% confidence interval (CI) 0.4–0.9). This may be attributed to PUFA's upregulation of anticancer defense, such as T-cell response [15, 16]. Additionally, a protective effect of vitamin D emerged (OR = 0.6, 95% CI 0.4–0.9). Though the ORs were for NHL overall, the author stated that these trends were especially true for FL. Erber et al. further studied vitamin D as a potential FL risk factor [17] in a study of 514 men and 425 women with FL. Subjects in the highest quartile of vitamin D intake had a 36% lowered risk than those in the lowest quartile (hazard ratio (HR) = 0.75, 95% CI 0.28–1.96 in men, HR = 0.64, 95% CI 0.29–1.41 in women), though these finding were not significant. 

Erber et al. also analyzed dietary patterns and risk of NHL subtypes, specifically diffuse large B-cell lymphoma (DLBCL) and FL [18]. Based on a calibrated quantitative FFQ and compared to a multiethnic cohort of 215,000 individuals from Hawaii, researchers used Factor Analysis to identify dietary patterns [19]. Variables were only incorporated if they had a factor loading greater than 0.55 and at least three variables loaded. The three loaded dietary patterns observed were “vegetable” (fruit, vegetables), “fruit and milk” (milk, yogurt, fruit), and “fat and meat” (meat, organ meat, frankfurters, sausage, luncheon meat, white potatoes, nonwhole grains, eggs, cheese). Men with a “fat and meat” dietary pattern (highest quartile versus lowest) had an increased risk of FL (HR = 5.16, 95% CI 1.33–20.0). In a Swedish study, Chang et al. found that fruit and vegetable intake was associated with decreased FL risk [20]. Vegetable intake was associated with a decreased FL risk (OR = 0.3, 95% CI 0.1–0.7). All vegetable subtypes also had OR ≤ 0.4 (cruciferous vegetables, green leafy vegetables, green vegetables, and red/orange vegetables). There was also an inverse association between FL risk and fruit intake (OR = 0.4, 95% CI 0.2–1.0).

Using Department of Agriculture databases, Frankenfeld et al. studied flavonoid intake with a 117-item FFQ [21]. Researchers acquired data on flavanol (tea, red wine), flavanone (citrus), flavone (fruit skins, peppers, leafy vegetables), isoflavone (soy foods), flavonol (leeks, onions, leafy vegetables, tomatoes), anthocyanidin (berries), and proanthocyanidin (chocolate, apples, nuts) intake. Increased flavonol (*P*-trend = 0.02), epicatechin (*P*-trend = 0.01), proanthocyanidin (*P*-trend = 0.02), and total flavonoid (*P*-trend < 0.01) intake was associated with decreased FL risk. Because previous studies had linked lower antioxidant intake to compromised immune systems [22–24], Thompson et al. analyzed the effect of antioxidant intake and FL risk [25]. Comparing 90 FL cases to a cohort of 35,159 Iowan women, FFQ data suggested inverse relationships between FL risk and vitamin C, lutein + zeaxanthin, *β*-cryptoxanthin, isoflavones, and flavonols (Table 1).

Kilfoy et al. conducted standardized personal demographic interviews and mailed FFQs to Connecticut women with FL (*n* = 134) in order to study the effects of nitrate and nitrite intake [26]. Increasing nitrate intake was positively associated with FL risk (*P*-trend = 0.04). Increasing overall nitrite intake from both plants (*P*-trend = 0.07) and animals (*P*-trend = 0.04) was also positively associated with FL risk (*P*-trend = 0.008). These studies suggest an association between FL and certain dietary habits that are stereotypical of the Western lifestyle. These dietary variables may account for some of the geographic and ethnic variability in FL. FL incidence patterns and their association with diet should be further explored.

### 2.2. Alcohol and FL

Alcohol is known to induce cancer in humans [27] and to lead to immunosuppression [28]. Casey et al. created a standard questionnaire-based interview and collected clinical and biological data from 298 lymphoma (34 FL) patients [29]. The questionnaire comprised a detailed life history of alcohol consumption and was also profession-specific. Wine consumption marginally increased the risk of FL (OR = 2.19, 95% CI 0.83–5.80, Table 4) with a higher risk for drinkers who started before the age of 20 years (OR = 4.04, 95% CI 1.19–13.76) and for drinkers who consumed more than 19 gm of alcohol per day (OR = 4.37, 95% CI 1.04–18.45). The researchers hypothesized that, with an increase in wine consumption, the risk decreases first and increases afterwards due to the exposure of resveratrol and ethanol respectively.

### 2.3. Smoking and FL

According to Morton et al., smoking was associated with increased risk estimates for FL (*n* = 1452) [30]. Compared to nonsmokers, current smokers had a higher risk for FL (OR = 1.31, 95% CI 1.12–1.52, Table 4) than former smokers (OR = 1.06, 95% CI 0.93–1.22). Current heavy smoking (>36 pack-years) was associated with a 45% increased OR for FL (OR = 1.45, 95% CI 1.15–1.82). While there was an increased risk of developing FL associated with cigarette smoking, the same was not true for other NHL subtypes. Troy et al. found cigarette smoking to be inversely associated with FL (ever smoking versus never smoking: HR = 0.62, 95% CI 0.45–0.85), but there was not a clear pattern [31]. The inverse association between smoking and FL persisted in analyses involving sex, age category, and level of education and after controlling for body mass index (BMI), drinking status, total number of drinks per week, grams of ethanol consumed per week, and several dietary factors. 

### 2.4. Anthropometrics and Body Mass Index

The anthropometric risk factors associated with FL are summarized in Table 3. In 2007, Larsson and Wolk performed a meta-analysis of studies examining the association between NHL and obesity [32]. Their study found an overall significant positive association (OR = 1.20, 95% CI 1.07–1.34), and this significance was maintained in their analysis of obesity and the diffuse large B-cell subtype, but disappeared in their analysis of FL. Their meta-analysis identified 6 studies, 5 of which were case-control studies, that specifically addressed the relationship between obesity and FL, and they estimated an overall OR of 1.10 (95% CI 0.82–1.47). Their meta-analysis was limited by the research designs of the included studies, a tendency towards measurement of obesity at a single time point, and possible publication bias. Nevertheless, this section discusses recent studies that have reexamined the association between anthropometrics, physical activity, and FL risk.

Maskarinec et al. looked at the relationship between body weight at different periods in a person's life and the effect on the incidence of NHL and its most common subtypes within a multiethnic cohort that included 129 cases of FL [33]. At the age of 21, BMI and body weight were significantly associated with NHL with HRs of 1.86 (95% CI 1.02–3.40) and 1.41 (95% CI 1.03–1.91), respectively. When stratified by subtype and gender, these variables resulted in incalculable or insignificant HRs as a result of the small sample sizes in men. The HRs for FL and the highest quartile of weight at age of 21 and height in men were 1.82 (95% CI 0.39–8.37) and 3.89 (95% CI 0.86–17.55). While these associations are not significant, they remain interesting clinical questions to explore in studies with a larger sample size. For women in the highest BMI category at cohort entry, the HR for FL was 6.16 (95% CI 1.75–21.71); however, the test for trend did not attain statistical significance (*P*-trend = 0.20). Other related measures including BMI at age of 21, weight at cohort entry, and weight at age of 21 were not associated with increased risk of FL. Data from this study suggest that the influence of weight and obesity, if any, on FL risk may be exerted early in life. However, this study does not support a clear association.

Another study by Chiu et al. [34] found that obesity between the ages 40–49 was associated with a higher risk of FL (OR = 1.8, 95% CI 0.9–3.5). The association between BMI and risk of NHL did not vary by gender. While these results were not statistically significant, they may still indicate a link between a high BMI and a higher chance of developing FL. The relationship between obesity and FL risk may also be confounded by dietary choices. Obese patients may consume more fat but fewer fruits and vegetables, both of which may be risk factors for NHL. Overall the data suggest that a higher BMI may be associated with a higher risk of NHL regardless of physical exercise and energy intake. 

Pan et al. conducted a population-based case control study of 1030 patients diagnosed with NHL (242 FL) to examine the impact of diet, energy intake, and physical activity [35]. Compared to patients in the lowest quartile of physical activity, the OR for FL for patients in the highest quartile was 0.64 (95% CI 0.42–0.97), while the OR for NHL in men was 0.79 (95% CI 0.59–1.05) and 0.59 (95% CI 0.42–0.81) for women. Physical activity has been associated with improved immune function and therefore may be associated with a decreased risk of NHL. Obesity has also been associated with decreased immune function. There was a higher risk reduction seen in women than in men. For FL, high caloric intake is associated with an increased risk, whereas physical activity is associated with a greater risk reduction.

Cerhan et al. conducted a study based on 4 SEER registries. Patients with a diagnosis of NHL who were between the ages of 20 and 74 were selected to be in the study [36]. Their diets, weight, and physical activity were examined using patient interviews. There is a positive association between height and a risk of FL. Patients in the tallest height category (71–78 inches) had an OR of 2 (95% CI 1.18–3.39; *P*-trend = 0.01). Nonoccupational physical activity was inversely associated with a risk of NHL and for FL specifically. There was no association found between type 2 diabetes, hypertension, or total energy intake. Troy et al. also analyzed the effect of anthropometry on FL risk. In both men and women, increased BMI was positively associated with NHL, with a higher risk for overweight and obese peoples compared to people with normal weight (*P*-trend < 0.01) [31]. Lifetime weight change was moderately associated with NHL even though no linear trend was detected. Greater height was shown as a risk factor only in men and not women (top quartile versus bottom: for men, HR = 1.46, 95% CI 1.13–1.88; for women, HR = 1.00, 95% CI 0.79–1.25; *P* for difference = 0.04). Risk estimates for anthropometric measures were higher for DLBCL and plasma cell neoplasms than for FL and small lymphocytic lymphoma/chronic lymphocytic leukemia. Overall, there is not enough evidence to conclude any definitive associations between obesity and FL risk. Interestingly, in two of the reviewed studies, height was significantly associated with FL in men but not in women. Our paper also suggests that lack of physical activity may be emerging as an independent risk factor for FL. 

### 2.5. Comorbid Medical Conditions and FL

NHL risk is associated with a variety of immunosuppressive states including HIV/AIDS, autoimmune diseases, and the immunosuppressive drug regimens that are used to treat autoimmune disease and transplant patients, highlighting the importance of comorbid disease and medical history as a factor in lymphoma risk [2]. While infection with Helicobacter pylori, Epstein-Barr virus, human herpes virus 8, human T-lymphotropic virus, and hepatitis-C have been implicated in the development of specific lymphoma subtypes, FL has not been definitively associated with any particular precursor medical condition.

Holly and Bracci performed a population-based case-control study in the San Francisco Bay Area from 1988 and 1995 analyzing the relationship between various NHL subtypes and aspects of the medical history [37]. In their multivariable age and sex-adjusted analysis, they found significant inverse associations between FL and nonsteroidal anti-inflammatory drug (NSAID) use (OR = 0.67, 95% CI 0.44–1.00), noninsulin diabetes mellitus treatment (OR = 0.29, 95% CI 0.1–0.82), a history of hepatitis (OR = 0.36, 95% CI 0.17–0.74), and three or more bee stings (OR = 0.79, 95% CI 0.62–1.00). The same analysis also showed positive association between FL and a history of heart disease (OR = 1.5, 95% CI 1.0–2.1) and *β*-blocker use (OR = 2.5, 95% CI 0.9–7.0). The investigators suggested that these conditions exert immunomodulatory effects that influence the development of NHL subtypes, a hypothesis that is consistent with the established relationship between immunosuppression and lymphoma incidence in other subtypes. Research showing that bee venom has anti-inflammatory effects in animal models and that *β*-blockers modulate immune cell signaling and tumor growth lends further biologic plausibility to their argument [38, 39]. Another study found that a history of multiple blood transfusions for medical conditions (rather than surgical or obstetric) was associated with NHL, but these findings did not reach statistical significance for FL [40]. This association may be confounded by underlying medical conditions that also increase the risk of NHL/FL or it may be related to the transmission of infectious agents that play a role in lymphomagenesis [40]. Studies have found that the hepatitis-B virus is associated with a risk of malignant lymphoma [41]. 

The InterLymph Consortium published a pooled analysis of individual data from 12 case-control studies looking at the risk of NHL in patients with a self-reported history of autoimmune disease [42]. They found an association between primary and secondary Sjogren's syndrome and FL with ORs of 1.78 (95% CI 0.34–9.37) and 7.55 (95% CI 1.75–32.7), respectively, and a pooled OR of 3.91 (95% CI 1.39–11.0). FL was not significantly associated with any other autoimmune disorders in this study. Lankes et al. also investigated vaccination history as a potential risk factor for FL in a case-control study of 387 NHL patients in Nebraska using telephone interviews that asked about demographics, date, and number of specific vaccinations [43]. Polio vaccination was associated with decreased risk (OR = 0.54, 95% CI 0.31–0.92) and influenza vaccination was associated with increased risk (OR = 1.98, 95% CI 1.23–3.18). The authors hypothesized that early exposure to antigens could alter the Th1 and Th2 immune responses and thereby influence FL risk [44]. However, the understanding of the relationships between specific vaccinations and FL risk remains incomplete.

The effect of allergies on FL risk has been debated. Some studies have found significant associations [45–51] but others have not [52–59]. In 2001, Briggs et al. found no evidence that a general history of allergy was associated with NHL overall (OR = 1.0, 95% CI 0.8–1.2) or for its major subtypes [60]. The research team also claimed that many of the previous NHL allergy studies (1970s to 2001) had found inconsistent or unreliable associations; many OR values were not adjusted for confounding factors and single measures of association were used to combine specific allergies into nonspecific or semirelated groups [48, 49, 51, 52, 55, 57–59]. In 2007, however, Cozen et al. analyzed 1,321 NHL cases and found a significant inverse association between allergy history and NHL overall, as well as for FL specifically (OR = 0.7, 95% CI 0.5–1.0) [61]. For eczema, on the other hand, there was a positive association with FL risk (OR = 1.9, 95% CI 1.1–3.4). The aspects of the medical history associated with FL are summarized in Table 2. The evidence to date on the associations between FL risk and aspects of the medical history including vaccination and autoimmune reactions raises the importance of immune regulation in the pathogenesis of FL and other NHL subtypes. However, additional research is needed to clarify these relationships. 

### 2.6. Environmental Exposures and FL

Purdue et al. looked at HIV-negative cases of NHL in 4 different SEER registries. UV exposure may reduce the risk of NHL [62]. The association between NHL and time spent in the sun within the last decade was dependent on the Ex11 + 32 T > C polymorphism in the vitamin D receptor gene. For FL, compared to individuals homozygous for the T allele who reported <7 hours per week of sun exposure, CC subjects with <7 hours per week of sun exposure had an OR of 6.3 (95% CI 1.9–22.0, p-interaction = 0.004). The biologic background of the UV exposure-NHL association may be due to vitamin D, given that the Vitamin D receptor is expressed in B and T-lymphocytes. 

 Many studies have examined the association between pesticide exposure and NHL, though the results have been inconsistent. In a case-cohort study that included 128 exposed farmers and 25 controls from the same region, Agopian et al. found a statistically significant association between exposure to pesticides and the development of the *t*(14, 18) translocation, a potential precursor lesion for *t*(14, 18)+ FL [63]. The frequency of the translocation increased by 87% in the control group and by 253% in the exposed group over a 9-year-period (*P* < 0.0001). Roulland et al. performed a cross-sectional study that found a higher prevalence and frequency of BCL2-IGH translocation amongst farmers during the high pesticide use period compared to farmers during the low pesticide use period, though these findings were not statistically significant (*P* = 0.10 and *P* = 0.12, resp.) [64]. The significance of these findings is unknown, as this translocation has been found in the peripheral blood of 50–80% of healthy individuals, most of whom never develop FL [7, 65, 66]. Their finding is consistent, however, with that of Chiu et al. who found a statistically significant increased risk of *t*(14, 18)+ NHL amongst farmers exposed to pesticides as compared to the unexposed, though this finding was not specific to the FL subtype [67]. Agopian et al. also looked at the clonality of *t*(14, 18) translocations by examining the variety of BCL2/*J*_*H*_  junctions in the exposed and unexposed. Individuals in both groups demonstrated surprisingly few variants of the translocation, suggesting that lymphomagenesis occurs through an immunogenic effect rather than by a genotoxic mechanism. 

According to a hospital-based case control study conducted by Wong et al., in Shanghai, low education level, living on a farm, home renovation, planting crops, and exposure to livestock were all associated with an increased risk of NHL [68]. None of these factors were significantly associated with FL risk in a subtype-specific analysis. FL risk was increased with the use of hair dyes (OR = 1.57, 95% CI 0.72–3.46), consistent with the findings of de Sanjose et al. (OR = 1.33, 95% CI 0.90–1.96) [69]. They also found associations with residence within 100 m of high voltage power lines (OR = 1.86, 95% CI 0.75–4.63) and a history of smoking (OR = 2.18, 95% CI 0.75–6.35), but these associations did not achieve statistical significance. 

Cocco et al. found that risk of FL significantly increased with exposure to chemical solvents such as benzene, toluene, xylene, (considered together as BTX), and styrene [70]. Another study found that exposure to chlorinated hydrocarbons increased the risk of FL [71]. This study examined workplace exposures to these chemicals. FL risk increased with exposure to BTX (*P* < 0.01), benzene (*P* < 0.04), toluene (*P* < 0.01), and xylene (*P* < 0.004). There were significant upward trends for FL associated with BTX and styrene. These results seem to indicate that solvents such as benzene cause lymphomagenesis and induce various translocations, long arm deletion of chromosome 6 and trisomy of chromosomes 2, 4, 6, 11, 12, 14, and 18, which are often seen in lymphoma patients. Although these associations need to be confirmed, industries should make efforts to reduce the addition of benzene to solvents and fuels since it may be linked to a risk of FL.

De Roos et al. conducted a case-control study in 4 SEER regions to examine the effect of proximity to industrial facilities and the risk of NHL [72]. Previous studies have found that there may be an association between industrial pollution and NHL. This study reviewed residential proximity to industrial facilities in the decade before diagnosis of NHL. There was an increased risk of NHL associated with living near a petroleum refinery for 10 years. For FL, OR was 1.3 (95% CI 0.5–3.8; *P*-trend = 0.58). There was also an increased risk of NHL associated with proximity to primary metal industries and for FL there was an association between risk and living within 2 miles of one (OR = 1.2, 95% CI 0.8–1.7). The study found that overall there was no association between living close to manufacturing facilities of any type and risk of NHL. These results were different from previous studies. Even though the study does not provide strong evidence towards an association between FL and living near an industrial facility, it still highlights the fact that there are risks associated with proximity to industrial facilities. The environmental exposure risk factors associated with FL are summarized in Table 4. 

### 2.7. Molecular Biology and FL Risk

Several studies have identified family history as a risk factor for NHL and for the FL subtype, suggesting that FL may have a genetic component [73–76]. Conde et al. performed a three-stage genome-wide association study (GWAS) that drew from a population-based case-control study of NHL in the San Francisco Bay Area, the Scandinavian Lymphoma Etiology follicular lymphoma case-control study, and 6 case-control studies that participated in the Interlymph Consortium [77]. The authors found an association between FL and single nucleotide polymorphisms (SNPs) in the major histocompatibility complex (MHC) region at 6p21.32, which encompasses the *HLA-DR* and *HLA-DQ* genes. In a meta-analysis combining data from the three stages, two variants, rs10484561 and rs7755224, had combined *P* values of 1.12 × 10^−29^ and 2.00 × 10^−19^, and ORs for FL of 1.95 (1.72–2.22) and 2.07 (95% CI 1.76–2.42), respectively. The two variants were also thought to form an extended haplotype, HLA-DRB1*0101-HLA-DQA1*0101-HLA-DQB1*0501, which had an OR of 2.07 (95% CI 1.40–3.06). This result adds to the previous identification of a susceptibility locus at the 6p21.32 region, encompassing the psoriasis susceptibility region 1 (PSORS1). 

Smedby et al. studied 430 patients with FL and 605 controls in Denmark and Sweden. They selected polymorphisms and haplotypes in three DNA repair genes, previously associated with skin cancer and DNA repair capacity, to assess their impact on risk of FL, including possible gene interaction with cigarette smoking and sun exposure [78]. Researchers genotyped 19 SNP in the *ERCC2*, *XRCC1*, and *XRCC3* genes. OR and 95% CI were calculated using unconditional logistic regression and haplotype associations were assessed with a global score test. The investigators did not observe any associations between variation in the *ERCC2* and *XRCC1* genes and FL risk. In *XRCC3*, increased risk of FL was suggested for rare homozygotes of three SNPs [Rs3212024: OR = 1.8 (95% CI 1.1–2.8); Rs3212038: OR = 1.5 (95% CI 1.0–2.4); Rs3212090: OR = 1.5 (95% CI 1.0–2.5)]. These results were strengthened in current cigarette smokers. However, evidence of differences in *XRCC3* haplotype distributions between FL cases and controls was weak, both overall and in current smokers. These findings are summarized in Table 2. Polymorphic variation in the *XRCC3* gene, but not in *ERCC2* or *XRCC1*, may be of importance for susceptibility to FL, perhaps primarily in current smokers. The link between skin cancer and FL is unlikely to be mediated through common variation in the studied DNA repair gene polymorphisms.

## 3. Conclusion

The increased incidence of NHL over the last three decades has provoked a wealth of epidemiologic data on the risk factors that may be contributing to this trend. FL, in particular, has often been a subject of this research. Our review of the literature covers an array of risk factors, some of which are modifiable, that may be associated with FL. Importantly, FL has been found to be more common in North America, the UK, and South Africa than in other regions of the world. The incidence of FL is not only lower in Chinese and Japanese populations, but also actually increases in individuals of Chinese or Japanese descent who were born in the US, suggesting that FL risk has an important environmental component [79].

Our paper found evidence of weak associations between FL and many aspects of the Western lifestyle; these include obesity, decreased physical activity, diets high in meat and milk, as opposed to fruits and vegetables, as well as the presence of nitrates in the diet. Protective factors in the diet included fruits and vegetables, polyunsaturated fatty acids, and a variety of antioxidants [14, 17–26]. These findings are supported by studies that have found associations between physical activity and improved immune function, as well as obesity and impaired immune function. Our paper also suggests that dietary vitamin D may be protective against FL [17]. Another study found that sun exposure in individuals who are homozygous for the Ex11 + 32 T > C allele is also protective against the development of FL, an effect that may be modulated through vitamin D signaling on B and T cells [62]. 

There were also associations with other aspects of the medical history. FL was significantly associated with primary and secondary Sjogren's syndrome but not with other forms of autoimmune disease [42]. A history of allergies was not consistently associated with the development of FL, although other immune modulators had variable effects on FL risk [60, 61]. Polio vaccination, NSAID use, noninjection diabetes mellitus treatment, and three or more bee stings were all protective against the development of FL, whereas influenza vaccine, the use of *β*-blockers, and a history of heart disease were all positively associated with the development of FL [37]. These findings emphasize the role of immune interactions throughout an individual's lifetime in modulating risk of FL and lymphoproliferative disease as well. 

A number of environmental exposures have also been associated with the development of FL. These include exposure to pesticides, hair dyes, industrial solvents such as benzene, toluene, xylene, styrene, and chlorinated hydrocarbons, as well as residential proximity to industrial facilities such as petroleum refineries and primary metal industries [68–70, 72]. FL was also weakly and inconsistently associated with exposure to alcohol and tobacco consumption. Alcohol was associated with an increased risk of FL, while the associations with tobacco consumption varied in direction and magnitude [30, 31, 68]. More significant than their contribution to the development of disease is their association with poor survival [80]. Tobacco, alcohol use, and obesity were all negatively associated with survival. 

 Genetic susceptibility may also play a role in mediating FL risk. Two variants of the MHC II region at 6p21.32, rs10484561 and rs7755224, as well as SNPs in the PSORS1 region, were found to increase the risk of FL in GWAS study [77]. These findings are consistent with the increased risk of FL in subjects with a family history of certain lymphoid neoplasms, as reported in several studies [73–76]. There is also evidence that genetic and environmental risk factors may interact to produce disease; certain polymorphisms of the DNA repair gene, *XRCC3*, may increase the risk of developing FL, an association that was especially pronounced in current smokers, and the risk associated with UV exposure may be dependent upon polymorphisms in the vitamin D receptor gene [62, 78]. Future studies should explore the interrelationships between genetic and environmental factors such as these in larger populations to improve our understanding of the factors underlying the development of FL.

## Figures and Tables

**Table 1:** Dietary risk factors associated with the development of follicular lymphoma.

Exposure	Measure of effect (95% CI)	*P*-trend	Reference
Diet			

Meat and milk	HR = 5.16 (1.33–20.0)	0.03	[19]
Fruits and vegetables	OR = 0.3 (0.1–0.7)	0.002	[20]
Vegetables^a^	OR = 0.3 (0.1–0.7)	0.001	[20]
Fruits^a^	OR = 0.4 (0.2–1.0)	0.04	[20]

Dietary factors			

Vitamin D	OR = 0.3 (0.1–0.9)	<0.05	[14]
Vitamin D*	HR = 0.64 (0.29–1.41)	0.38	[17]
PUFA	OR = 0.4 (0.2–1.1)		[14]
Nitrate (mg/day)	OR = 1.5 (0.8–2.7)	0.04	[26]
Nitrite (mg/day)	OR = 2.3 (1.1–4.9)	0.008	[26]

Antioxidants			

Vitamin C	RR = 0.55 (0.30–1.0)	0.032	[25]
Lutein + zeaxanthin	RR = 0.56 (0.32–0.99)	0.007	[25]
B-cryptoxanthin	RR = 0.57 (0.31–1.05)	0.04	[25]
Isoflavones	RR = 0.53 (0.29–0.97)	0.022	[25]
Flavonols	RR = 0.52 (0.29–0.94)	0.03	[25]

^a^These odds ratios are for the female cases only.

**Table 2:** Aspects of the medical history and genetic factors associated with FL.

Exposure	Measured effect (95% CI)	Reference
Medical Hx		

Hepatitis history	OR = 0.36 (0.17–0.74)	[37]
History of heart disease	OR = 1.5 (1.0–2.1)	[37]
3 or more bee stings	OR = 0.79 (0.62–1.0)	[37]
Gallstones	OR = 0.71 (0.33–1.52)	[36]
Hypertension	OR = 0.92 (0.63–1.94)	[36]
DM, no treatment	OR = 2.53 (0.57–11.2)	[36]
Sjogren's syndrome	OR = 3.91 (1.39–11.0)	[42]
History of transfusion	OR = 1.40 (0.87–2.26)	[40]
Medical indication for first transfusion (ref = Trauma)	OR = 3.07 (1.18–7.98)	[40]
Any anesthesia (24+)	OR = 1.49 (0.76–2.91)	[40]
Total number of surgeries (26+)	OR = 1.92 (0.97–3.78)	[40]

Pharmaceuticals and vaccinations		

Polio vaccination	OR = 0.54 (0.31–0.92)	[43]
Influenza vaccination	OR= 1.98 (1.23–3.18)	[43]
NSAID use	OR = 0.67 (0.44–1.00)	[37]
Non-insulin diabetes mellitus treatment	OR = 0.29 (0.1–0.82)	[37]
Beta blockers	OR = 2.5 (0.9–7.0)	[37]
DM, no treatment	OR = 2.53 (0.57–11.2)	[36]
DM, oral treatment	OR = 0.46 (0.61–1.35)	[36]
DM, insulin	OR = 0.63 (0.14–2.92)	[36]
Calcium channel blocker use	OR = 1.08 (0.60–1.94)	[36]
Diuretic use	OR = 0.83 (0.48–1.44)	[36]

Allergies		

General Hx of allergies	OR = 0.7 (0.5–1.0)	[61]
Hay fever	OR = 0.7 (0.5–1.1)	[61]
Eczema alone	OR =1.9 (1.1–3.4)	[61]

Molecular		

*SNPs within the MHC region, 6p21.32*		
rs10484561	OR = 1.95 (1.72–2.22)	[77]
rs7755224	OR = 2.07 (1.76–2.42)	[77]
Haplotype HLA-DRB1* 0101-HLA-DQA1*0101-HLA-DQB1* 0501	OR = 2.07 (1.40–3.06)	[77]
*SNPs within DNA repair genes*		
ERCC2	No association	[78]
XRCC1	No association	[78]
XRCC3_Rs3212024	OR = 1.8 (1.1–2.8)	[78]
XRCC3_Rs3212038	OR = 1.5 (1.0–2.4)	[78]
XRCC3_Rs3212090	OR = 1.5 (1.0–2.5)	[78]

**Table 3:** Anthropomorphic factors associated with the development of follicular lymphoma.

Exposure	Measure of effect (95% CI)	*P*-trend	Reference
BMI			

BMI > 30	RR = 1.10 (0.82–1.47)		[32]
BMI > 30 at cohort entry (men)	HR = 1.86 (0.44–7.86)	0.09	[33]
Usual adult BMI > 30	OR = 1.4 (0.8–2.5)	0.2	[34]
Baseline BMI > 30	HR = 1.03 (0.67–1.60)	0.465	[31]

Weight			

Highest weight category at cohort entry	HR = 3.20 (0.74–13.84)	0.18	[33]
Highest weight category at age of 21 (men)	HR = 1.82 (0.39–8.37)	0.47	[33]
Highest category of weight	OR = 0.66 (0.31–1.41)	0.2	[36]
Highest category weight at age of 20 years	HR = 1.32 (0.83–2.10)	0.182	[31]
Highest category of baseline weight	HR = 1.14 (0.72–1.82)	0.555	[31]
>6 kg weight gain/10 years	HR = 1.16 (0.71–1.90)	0.946	[31]
Highest category of positive annual weight gain	HR = 5.34 (0.89–32.06)	0.24	[33]

Height			

Highest category at cohort entry (men)	HR = 3.89 (0.86–17.55)	0.09	[33]
Highest category of Height	OR = 1.28 (0.56–2.93)	0.6	[36]
Highest category of baseline height	HR = 1.07 (0.67–1.71)	0.571	[31]

Calorie intake			

Highest category of total cal. intake	OR = 1.69 (1.14–2.51)	0.002	[35]
Highest Category of total energy intake	OR = 0.82 (0.45–1.52)	0.8	[36]

Physical activity metabolic equivalents			

Highest cat. of mod phys. act.	OR = 0.79 (0.53–1.19)	0.16	[35]
Highest cat. of vigorous phys. Act.	OR = 0.59 (0.38–0.91)	0.037	[35]
Highest cat. of tot. phys. act.	OR = 0.64 (0.42–0.97)	0.042	[35]
Highest cat. of Nonoccupational phys. act.	OR = 0.79 (0.40–1.56)	0.5	[36]

**Table 4:** Environmental and lifestyle exposures associated with follicular lymphoma.

Exposure	Measure of Effect (95% CI)	*P*-trend	Reference
Alcohol			

Highest category of beer consumption (drinks/week)	HR = 0.90 (0.40–1.99)	0.553	[31]
Highest category of wine consumption (drinks/week)	HR = 0.96 (0.49–1.90)	0.513	[31]
Highest category of liquor consumption (drinks/week)	HR = 0.71 (0.30–1.69)	0.395	[31]
Highest category of alcohol consumption (drinks/week)	HR = 0.71 (0.28–1.79)	0.302	[31]
Highest category of ethanol consumption (g/week)	HR = 0.55 (0.24–1.30)	0.295	[31]
Wine	OR = 2.19 (0.83–5.80)		[29]
Alcohol use (ever/never)	OR = 1.22 (0.51–2.89)		[68]
Survival: alcohol use <43.1 g/weeka	HR = 0.35 (0.10–1.20)		[80]
Survival: alcohol use >43.1 g/week	HR = 2.16 (0.98–4.77)		[80]

Tobacco			

Ever smoker	HR = 0.62 (0.45–0.85)		[31]
Current smoker	HR = 0.74 (0.42–1.31)		[31]
Former smoker	HR = 0.59 (0.42–0.83)		[31]
>30 years since quitting (former smokers)	HR = 0.42 (0.21–0.84)	0.374	[31]
Age at initiation (>20)	HR = 0.80 (0.47–1.34)	0.318	[31]
>40 years of having smoked	HR = 0.52 (0.28–0.97)	0.365	[31]
Packs of cigarettes/day	HR = 0.59 (0.32–1.09)	0.627	[31]
Pack years of smoking >39.75	HR = 0.63 (0.37–1.07)	0.949	[31]
Ever smoker	OR = 1.15 (1.02–1.29)		[30]
Current smokers	OR = 1.31 (1.12–1.52)		[30]
Former smokers	OR = 1.06 (0.93–1.22)		[30]
Ever smokers >36 pack years	OR = 1.30 (1.08–1.56)	0.6	[30]
Current heavy smokers > 36 pack years	OR = 1.45 (1.15–1.82)	0.85	[30]
Survival: former smoker	HR = 1.98 (0.81–4.80)		[80]
Survival: current smoker	HR = 2.95 (1.27–6.81)		[80]
Survival: >17 pack-years	HR = 2.61 (1.19–5.74)	0.02	[80]
Survival: time since quitting to diagnosis (<10 years versus never Smoker)	HR = 3.73 (1.07–13.0)	0.005	[80]
ever smoker	OR = 2.18 (0.75–6.35)		[68]

Environmental exposures			

UV exposure and Ex11 + 32 CC genotype: <7 hrs/wk sun exposure in last 10 years	OR = 6.3 (1.9–22.0)	0.004, *P*–interaction = 0.009	[62]
UV exposure and Ex11 + 32 CC genotype: <7 hrs/wk sun exposure in the fourth decade	OR = 5.7 (1.5–21)	0.01, *P*-interaction = 0.003	[62]
UV exposure and Ex11 + 32 CC genotype: <7 hrs/wk sun exposure in the third decade	OR = 8.3 (1.9–37)	0.03, *P*-interaction = 0.03	[62]
UV exposure and Ex11 + 32 CC genotype: <7 hrs/wk sun exposure in the second decade	OR = 20.0 (1.4–304)	0.06, *P*-interaction = 0.05	[62]
Hair dyes	OR = 1.33 (0.9–1.96)		[69]
Hair dyes	OR = 1.57 (0.72–3.46)		[68]
All solvents	OR = 1.3 (1.0–1.8)	0.03	[70]
BTX exposure (benzene, toluene, xylene)	OR = 1.6 (1.0–2.5)	0.08	[70]
Benzene	OR = 1.6 (0.9–2.9)	0.1	[70]
Toluene	OR = 1.5 (0.9–2.4)	0.2	[70]
Xylene	OR = 1.6 (0.9–2.7)	0.14	[70]
CAH exposure	OR = 1.1 (0.6–1.9)	0.9	[70]
Trichloroethylene	OR = 1.2 (0.6–2.3)	0.65	[70]
Gasoline exposure	OR = 0.8 (0.5–1.5)	0.3	[70]
Chlorinated hydrocarbons, total > 47.3 ppm∗years	OR = 3.9 (1.3–12.1)	0.04	[71]
Trichloroethene > 35 ppm∗yrs	OR = 3.2 (0.8–12.9)	0.16	[71]
Tetrachloroethene > 0, <9.1	OR = 1.2 (0.3–5.5)	0.43	[71]
CTET > 23, <48.1 ppm∗yrs	OR = 2.1 (0.2–19.4)	0.83	[71]
DCM > 26.3, <175 ppm∗yrs	OR = 1.5 (0.3–7.4)	0.2	[71]
Benzene > 130 ppm∗yrs	OR = 1.3 (0.3–6.4)	0.51	[71]
Toluene > 207 ppm∗yrs	OR = 1.1 (0.2–5.3)	0.48	[71]
Xylene > 230 ppm∗yrs	OR = 1.3 (0.3–6.2)	0.33	[71]
Styrene > 67.1 ppm∗yrs	OR = 1.6 (0.5–6.0)	0.2	[71]
Lived within 2 miles of a lumber and wood products facility^b^	OR = 1.1 (0.5–2.2)	0.84	[72]
Lived within 2 miles of a chemicals and allied products facility^b^	OR = 1.2 (0.8–1.8)	0.34	[72]
Lived within 2 miles of a petroleum refining and related industries^b^	OR = 1.1 (0.7–1.9)	0.89	[72]
Lived within 2 miles of a primary metal industries facility^b^	OR = 1.2 (0.8–1.7)	0.21	[72]
Lived within 100 m of high voltage power lines	OR = 1.86 (0.75–4.63)		[68]

^a^The reference group consumes 0 g/week.

^b^Ptrend is for half-mile increments within two miles proximity.
